# Functional significance of some common oxytocin receptor SNPs involved in complex human traits

**DOI:** 10.1186/s12860-024-00529-1

**Published:** 2025-01-06

**Authors:** Suk Ling Ma, Michael Thomas Bowen, Mark R. Dadds

**Affiliations:** 1 Department of Psychiatry, Faculty of Medicine, The Chinese University of Hong Kong, Hong Kong SAR, China; 2 Faculty of Science, School of Psychology, University of Sydney, Sydney, NSW Australia; 3 Brain and Mind Centre, University of Sydney, Sydney, NSW Australia

**Keywords:** Oxytocin, Polymorphisms, Functional significance

## Abstract

**Background:**

Oxytocin function is associated with a range of human traits and is often indexed by common polymorphisms of the receptor gene OXTR. Little is known however about the functional significance of these polymorphisms.

**Objectives:**

To examine the effects of common polymorphisms of OXTR on transcription expression in human neural cells.

**Method:**

The impact of four common OXTR SNPs (rs1042778, rs4686302, rs2254298 and rs237887) on OXTR gene expression were tested in human neuroblastoma cell line, SH-SY5Y, a commonly used cell line for neurological disease. SNPs were chosen as having robust evidence for associations with complex human traits after consideration of linkage patterns across OXTR.

**Results:**

The expression level of GG genotype of rs1042778 was significantly lower than TT genotypes. None of the other SNPs were related to functional transcription.

**Conclusions:**

OXTR polymorphisms showing robust associations with complex human traits are not reliably associated with changes in transcription of OXTR. Increasing cooperation between behavioral and biological scientists is needed to bridge the gap between human trait and functional biological studies to improve our understanding of oxytocin and other important mammalian neuroendocrine processes.

**Supplementary Information:**

The online version contains supplementary material available at 10.1186/s12860-024-00529-1.

## Introduction

Oxytocin (OXT) is a neuropeptide synthesized in the supraoptic and paraventricular nuclei of the hypothalamus. OXT is released from the posterior pituitary and acts in the periphery to regulate several important physiological functions, including stimulating uterine contractions during childbirth and controlling the milk letdown reflex in response to infant suckling. However, OXT is also released into numerous brain regions, where it plays an important role in regulating social and emotional behaviors [1]. The effects of endogenous OXT are mediated via its actions at the G protein-coupled OXT receptor (OXTR), which is expressed abundantly throughout the brain [2].

Recent research has shown that variations in OXT function are associated with individual differences in complex psychological and social functioning in humans [3]. Indexed in terms of circulating levels, and genetic and epigenetic variations in the OXTR gene, OXT function is associated with risk for psychopathologies involving social-interpersonal functioning such as autism spectrum disorder (ASD), attention deficit hyperactivity disorder (ADHD) and other childhood psychiatric disorders [4, 5], as well as individual differences in complex traits and behaviors such as empathy, pair bonding, and mutual eye gaze [6, 7].

Associations with complex traits and psychopathology have been established for several common polymorphisms of the OXTR gene [8]. For example, rs1042778 has been linked to ASD [9], as well as antisocial behavior and amygdala reactivity to emotional facial expressions [10]. SNP rs4686302 has been associated with empathy [11] and social cognitive deficits in children with ADHD [12]. SNPs rs2254298 and rs237887 are both associated with ASD [13, 14].

The typical research design for these studies has involved comparing frequencies of the common versus minor alleles in samples with and without disorders or with varying levels of complex traits of interest. Despite these associations, very little is known about how these alleles influence OXTR expression and function, which could provide important insights into how these SNPs might affect complex social and emotional behaviors and processes. One recent study reported the A218T variant of the OXTR that results from SNP rs4686302 altered intracellular signaling compared to WT OXTRs when expressed in HEK293 cells [15].

This supports the need for further examination of the disease- and complex trait-associated OXTR SNPs to elucidate cellular mechanisms that might facilitate a more causal understanding of their effects on social and emotional processes.

**Fig. 1:**
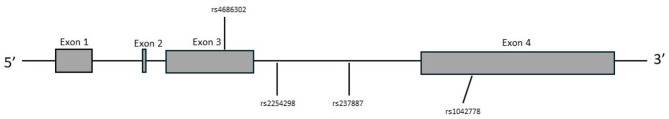
Figure showing the schematic gene structure and OXTR and the SNPs investigated in this study

Here we examined the impact of four common OXTR SNPs (rs1042778, rs4686302, rs2254298 and rs237887) on OXTR gene expression (Fig. 1). These were chosen as having the most robust evidence (at the beginning of our study in 2019) for associations with complex human traits. It should be noted that rs53576 is one of the most investigated SNPs for complex human traits; however, this SNP is located in intronic region and it is not expected to be contributed to the protein function or configuration of protein. Therefore, we searched for other SNPs which are in complete linkage disequilibrium (LD) with this SNP in the coding region for functional significance. rs4686302 is in complete LD with rs53576 and it’s a missense mutation (can lead to amino acid change), thus it was assayed in place of rs53576.

## Materials and methods

Four gene regions comprising four SNPs (rs1042778, rs4686302, rs2254298 and rs237887) of OXTR with each region spanning around 1200 bp were amplified from human genomic DNA by PCR using primers listed in Table 1. The PCR products containing the gene fragment were digested by the corresponding restriction enzyme as listed in Table 1. The fragment was then cloned to pGL3-Basic firefly luciferase expression vector (Promega, Madison, WI). Site-directed mutagenesis was performed using the QuikChange site-directed mutagenesis kit (Stratagene, La Jolla, CA) according to manufacturer’s instructions. This introduced the wild type and mutant type constructs for these SNPs. For rs1042778, both mutants T and A were reported in literature and dbSNP, therefore both mutant constructs were prepared accordingly. The sequences of the constructs were verified by Sanger sequencing to confirm the sequence of recombinant plasmid was correct without base mutation and deletion.

**Table 1:** Primers used in this study

Name	Sequence (5’- 3’)	Restriction enzyme
rs1042778F	ATCACTAGTTCCACGGCGTGACCCACCA	SpeI
rs1042778R	AACAAGCTTTACCGCTTTTCACAATATC	HindIII
rs4686302F	ATCGGTACCGGTGGACCCAGCAGATCCG	KpnI
rs4686302R	GATAGCTAGCCTTCCTTGGGCGCGTTGGCAT	NheI
rs2254298F	ATCGGTACCTGAACAGTCTTTGGCGTGTG	KpnI
rs2254298R	GATAGCTAGCTGCTAGGCCTGTACCCAAAA	NheI
rs237887F	ATCGGTACCCGGAAGGATTGGTGGCTACT	KpnI
rs237887R	GATAGCTAGCAAATGCACATTCTCAGGCCC	NheI
rs1042778mutTF	AGTTTGTATCCCTCCTCTCCTTGGGGTGGC	
rs1042778mutTR	GCCACCCCAAGGAGAGGAGGGATACAAACT	
rs1042778mutCF	AGTTTGTATCCCTCCGCTCCTTGGGGTGGC	
rs1042778mutCR	GCCACCCCAAGGAGCGGAGGGATACAAACT	
rs4686302mutF	CATCGTGCTCGCTACCTGCTACGGCCTTAT	
rs4686302mutR	ATAAGGCCGTAGCAGGTAGCGAGCACGATG	
rs2254298mutF	GAAACCATCCCTGTTTTCTCAGTTTGCGGGGCTTCTTC	
rs2254298mutR	GAAGAAGCCCCGCAAACTGAGAAAACAGGGATGGTTTC	
rs237887mutF	CATCCCTGATGCTTCCTTCTACCTCATTGCAAAGC	
rs237887mutR	GCTTTGCAATGAGGTAGAAGGAAGCATCAGGGATG	

Human neuroblastoma cell line, SH-SY5Y, is a commonly used cell lines for neurological disease. SH-SY5Y were cultured and cells were co-transfected with pGL3-basic vector construct cloned with the gene regions of interest containing the wild type or mutant and Renilla luciferase pRL-SV40 vector by using Lipofectamine 2000. Cells were harvested 48 h after transfection and lysed in lysis buffer. For results showing significant difference between wild-type and mutant, the plasmid and miRNA mimics were co-transfected to compare the effect of binding to miRNA. The pGL3-basic vector only has the luciferase gene and it was ligated to different gene fragment as listed in Table 1. This vector is useful in the study of functional promoter elements to regulate gene expression. Renilla luciferase pRL-SV40 vector was used to normalize and reduce differences in transfection efficiencies and subsequent variations in these experiments. Firefly and Renilla luciferase signals were measured by the Dual-Luciferase Reporter Assay System according to manufacturer’s instruction (Promega). Firefly luciferase activities were normalized to Renilla luciferase activity as “relative luciferase activity”. The measurement was performed in triplicates. The difference in the levels of luciferase assay between wild type and mutants were determined by Student’s t-test and One-way ANOVA analysis (SPSS 26.0) [16]. To confirm the result, another cell line, H4, which is epithelial cells from neuroglioma patient, was used to replicate all the experiments.

## Results

**Fig. 2:**
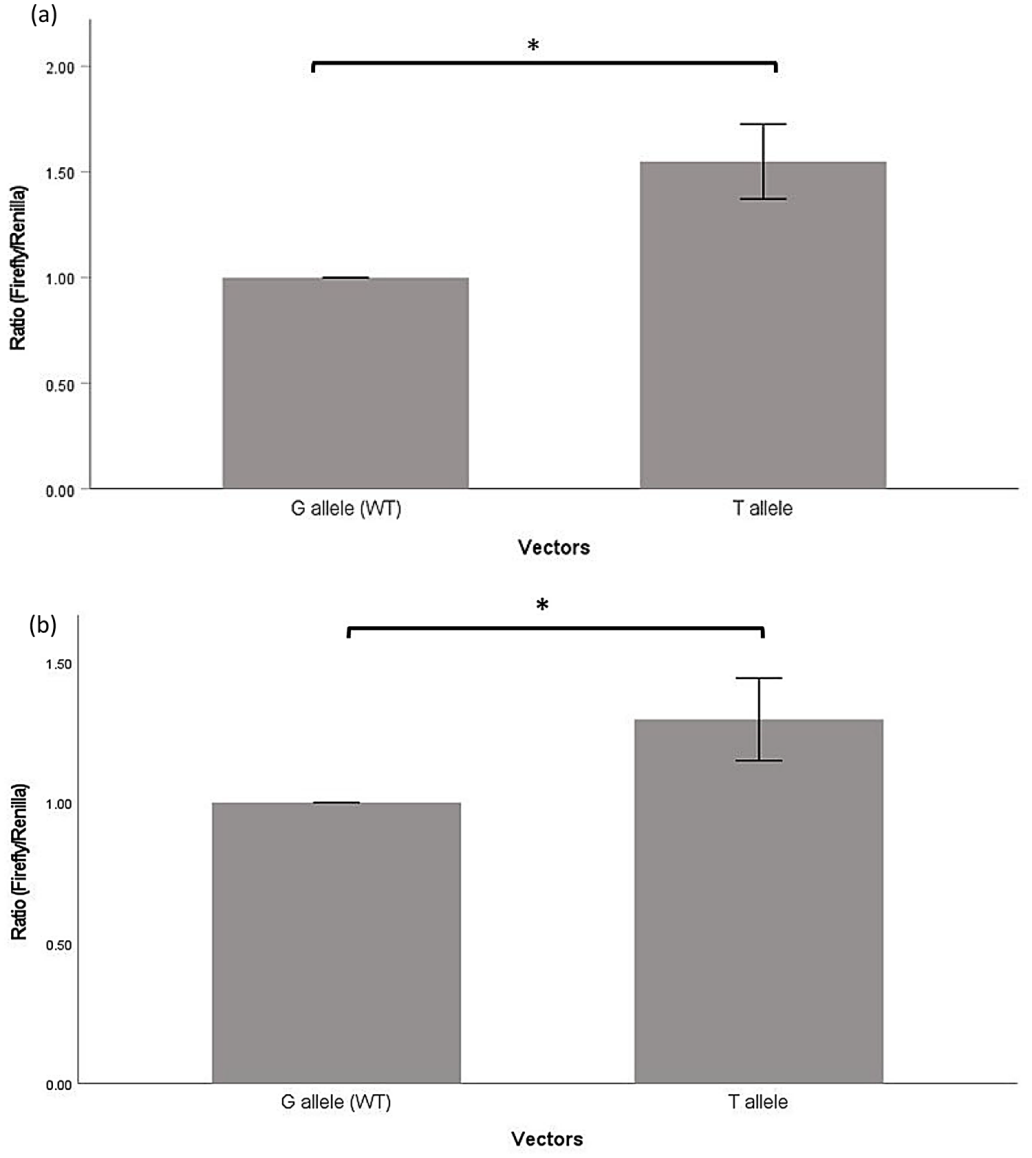
Luciferase reporter gene assay showing the effect of different alleles of SNP rs1042778 in **a** SH-SY5Y (*p* = 0.006) and **b** H4 (*p* = 0.013). * *p* < 0.05

**Fig. 3:**
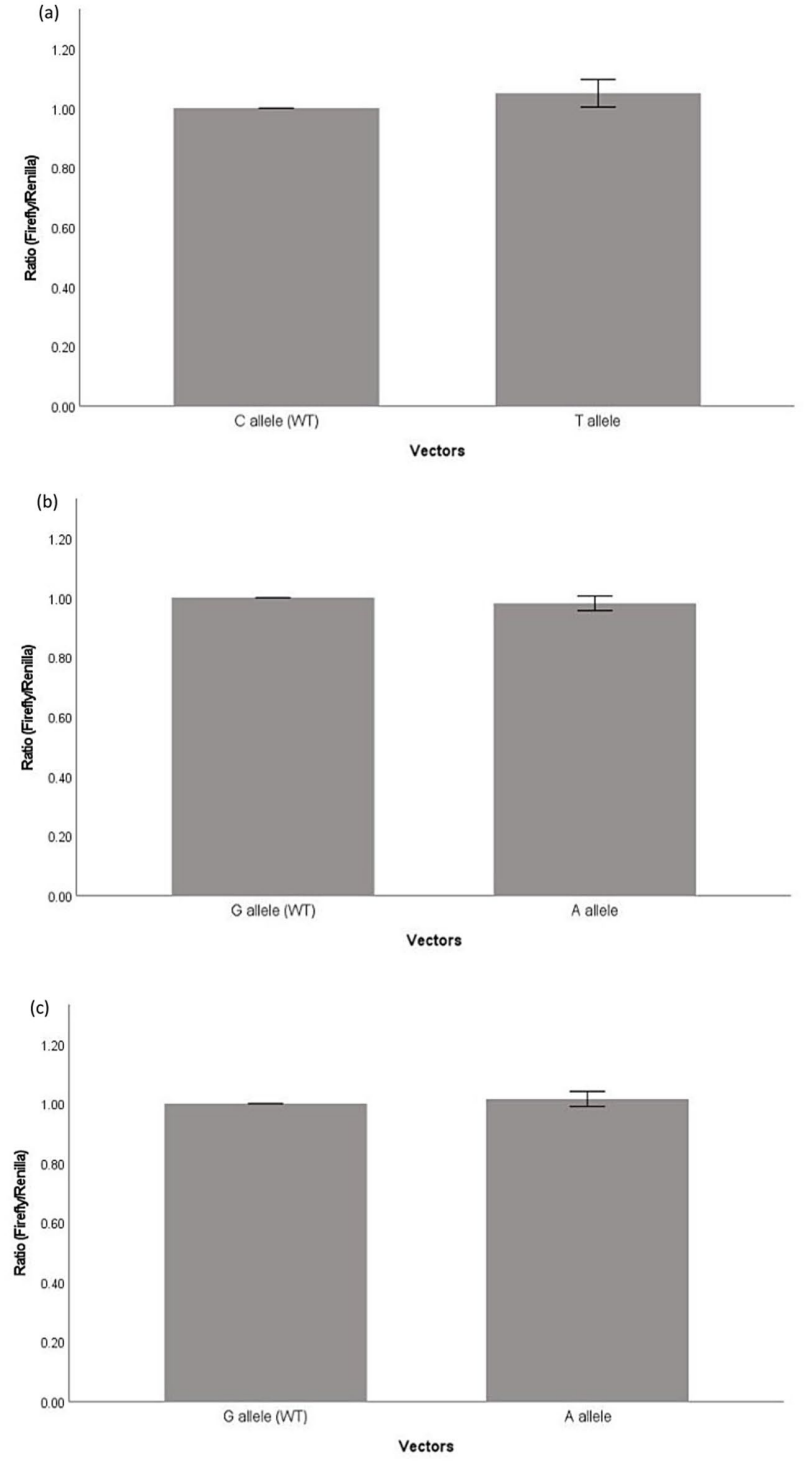
Luciferase reporter gene assay showed no significant difference between different alleles of **a** rs4686302 (*p* = 0.065); **b** rs237887 (*p* = 0.114) and **c** rs2254298 (*p* = 0.16)

Luciferase reporter assays were performed with reporter vectors containing four SNPs (rs1042778, rs4686302, rs2254298 and rs237887) wild type or mutant respectively, to investigate if the change of nucleotide on the SNPS affect the expression of OXTR. Our result showed the luciferase activity of rs1042778G (wild-type) was significantly lower than rs1042778T (*p* = 0.006) (Fig. 2a). For other SNPs (rs468302, rs2254298 and rs237887), there was no significant difference in the luciferase activity (Fig. 3). All the luciferase reporter assays testing the functional effects of these four SNPs was replicated and confirmed in another cell line, H4 (Fig. 2b and Suppl Fig. 1a). Bioinformatics analysis was performed to identify the SNPs in complete LD with the test SNPs (rs468302, rs2254298 and rs237887). The result showed these SNPs were in introns and therefore they are not expected to be functional (Suppl file).

**Fig. 4:**
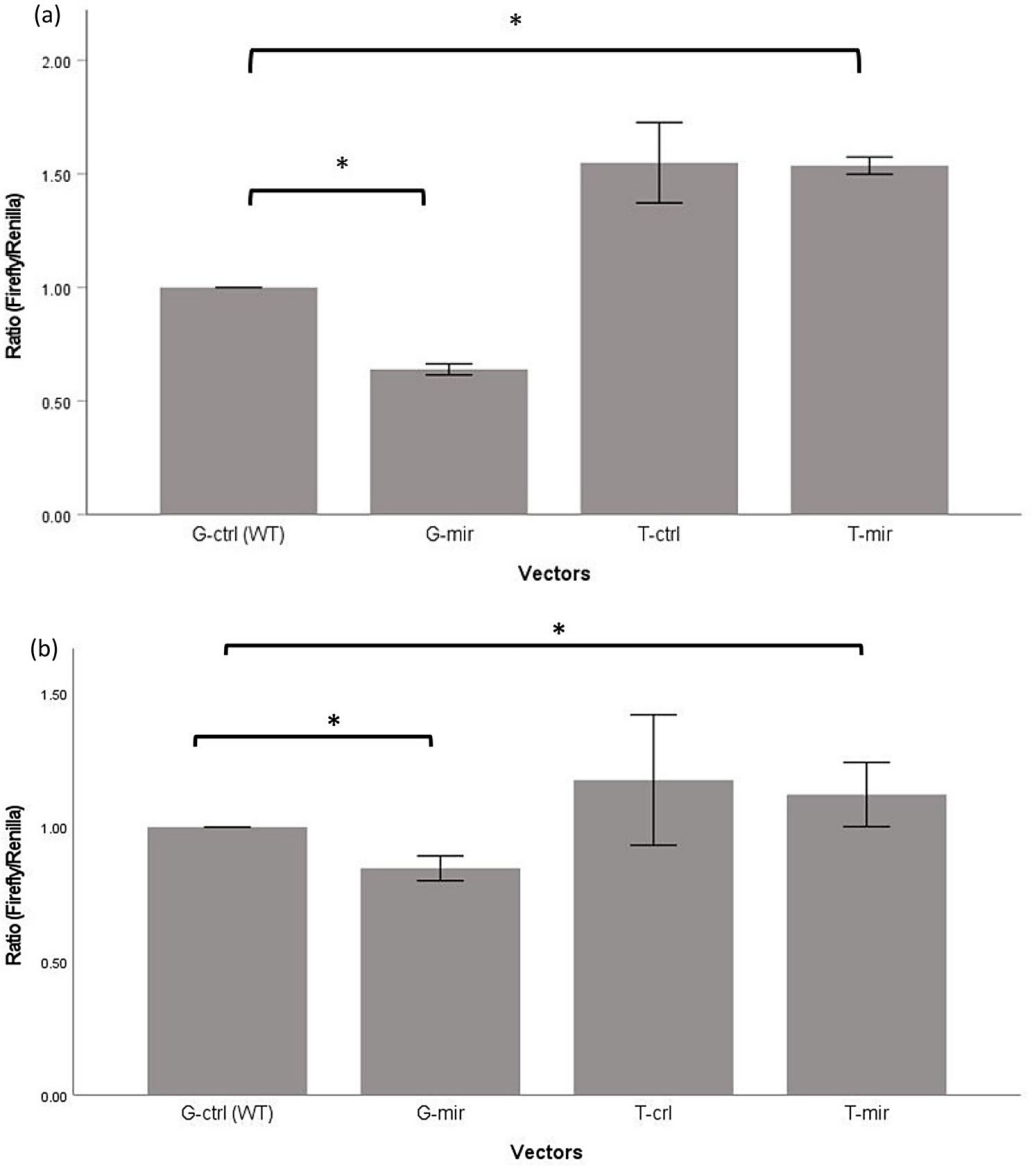
Luciferase reporter gene assay showing the effect of base substitution due to rs1042778 on miRNA binding in **a** SH-SY5Y and **b** H4. *Remarks ctrl* control miRNA, *mir* hsa-miR-29c mimics. * *p* < 0.05

Since there was significant difference on luciferase activity between G allele and T allele of rs1042778, bioinformatics analysis was performed and identified hsa-miR-29c is the possible miRNA binding site associating with this SNP. When hsa-miR-29c mimics was co-transfected with the reporter construct, significant suppression of luciferase activity was observed in constructs containing G (wild-type) alleles compared with the construct containing T allele, which presumably does not bind miRNAs (Fig. 4a). Our finding suggested that T allele of rs1042778 disrupted the miRNA binding and the result was confirmed by an independent cell line, H4 (Fig. 4b).

## Discussion/Conclusion

Considerable interest and research are being devoted to understanding the role of oxytocin in complex human traits. One way of characterizing OXT function that has received considerable support is correlating variations in OXTR polymorphisms with complex human traits [6, 14]. The function significance of these polymorphisms has received little attention in comparison. Here we looked at associations between common polymorphisms of the OXTR gene and transcription signal expression using luciferase reporter assays, in a human neural cell line known to show cellular changes associated with complex traits. Our findings showed that the expression level of GG genotype of rs1042778 was significantly lower than TT genotypes. rs1042778 was associated with different traits, such as callous–unemotional (CU) trait [17] and ASD.

However the findings for this SNP were the exception and none of the other SNPs showed any relation to functional transcription. This is somewhat surprising given the wealth of evidence relating these SNPs to human traits. First, we will canvass possible reasons for the null results. It is possible but highly unlikely that the human cell line we used does not manifest polymorphic driven changes in transcription signal for OXTR. OXTR expression is widespread in the human neural systems and this cell line has a positive history of being used to detect such changes. Second, it is possible that these SNPs are actually not functional but are in linkage with other SNPs that were not assayed in this study and were not measured in previous studies of human traits. For rs1042778, there are no SNPs with D’ over 0.12, suggesting it is in low linkage with other OXTR SNPs. However, rs4686302 is in complete LD with several SNPs such as rs53576, rs237902, rs237900, rs237899, and rs34880121; rs2254298 is in complete LD with rs2254295, rs237889, rs1131148 and other OXTR SNPs, and rs237887 is in complete LD with rs918316, rs4686301, and rs2268491. However, these SNPs fall in intron and might not be functional. Future research will need to complete a more comprehensive assay of OXTR SNPs to narrow down sources of functional change in transcription expression [18].

Third, recent decades have seen exponential growth in our understanding of the complexity of gene-transcription-trait relationships [19] and it is possible that these polymorphisms, if reliably functional, are operating through mechanisms other than direct changes to gene transcription and expression. Likely candidates would be by influencing the functional expression of other polymorphisms, and epigenetic processes such as methylation of the OXTR. For example [17, 20], showed that polymorphisms and methylation of the OXTR gene were related to low interpersonal empathy in children.

Notwithstanding the null findings for 3 of our chosen SNPs, we did show that polymorphisms of rs1042778 are associated with changes in transcription signal of OXTR. Previous research has been contradictory on which allele was functional for this SNP and our results show the functional T-type allele to be associated with lowered expression of OXTR. Given this SNP is not linked to other OXTR SNPs, it should be considered a candidate for changes to transcription expression of OXTR associated with complex human traits.

Overall these results show that the relationship of polymorphisms of the OXTR gene known to predict variations in complex human traits are likely to operate through varied mechanisms of influence, and only for the minority of SNPs will that be by directly influencing gene expression in neural cell lines. Limitations of our study include only focusing on a limited set of OXTR polymorphisms and limiting our analyses to direct transcription expression. Our results show that increasing cooperation is needed between behavioral scientists and cell biologists to bridge the existing gap between human trait and functional biological studies in our understanding of oxytocin and other important mammalian neuroendocrine processes.

## Electronic supplementary material

Below is the link to the electronic supplementary material.

Supplementary Material 1

Supplementary Material 2

Supplementary Material 3

## Data Availability

All data generated or analyzed during this study are included in this article. Further enquiries can be directed to the corresponding author.

## References

[1] Olff M, Frijling JL, Kubzansky LD, Bradley B, Ellenbogen MA, Cardoso C, et al. The role of oxytocin in social bonding, stress regulation and mental health: an update on the moderating effects of context and interindividual differences. Psychoneuroendocrinology. 2013;38(9):1883–94. 23856187 10.1016/j.psyneuen.2013.06.019

[2] Jurek B, Neumann ID. The oxytocin receptor: from intracellular signaling to behavior. Physiol Rev. 2018;98(3):1805–908. 29897293 10.1152/physrev.00031.2017

[3] Clarke L, Zyga O, Pineo-Cavanaugh PL, Jeng M, Fischbein NJ, Partap S, et al. Socio-behavioral dysfunction in disorders of hypothalamic-pituitary involvement: the potential role of disease-induced oxytocin and vasopressin signaling deficits. Neurosci Biobehav Rev. 2022;140:104770. 35803395 10.1016/j.neubiorev.2022.104770 PMC10999113

[4] Parker KJ, Garner JP, Libove RA, Hyde SA, Hornbeak KB, Carson DS, et al. Plasma oxytocin concentrations and *OXTR* polymorphisms predict social impairments in children with and without autism spectrum disorder. Proc Natl Acad Sci USA. 2014;111(33):12258–63. 25092315 10.1073/pnas.1402236111 PMC4143031

[5] Ayaz AB, Karkucak M, Ayaz M, Gokce S, Kayan E, Güler EE, et al. Oxytocin system social function impacts in children with attention-deficit/hyperactivity disorder. Am J Med Genet B Neuropsychiatr Genet. 2015;168(7):609–16. 26174935 10.1002/ajmg.b.32343

[6] Barchi-Ferreira AM, Osório FL. Associations between oxytocin and empathy in humans: a systematic literature review. Psychoneuroendocrinology. 2021;129:105268. 34023733 10.1016/j.psyneuen.2021.105268

[7] Pohl TT, Young LJ, Bosch OJ. Lost connections: Oxytocin and the neural, physiological, and behavioral consequences of disrupted relationships. Int J Psychophysiol. 2019;136:54–63. 29330007 10.1016/j.ijpsycho.2017.12.011 PMC6037618

[8] Kohlhoff J, Cibralic S, Hawes DJ, Eapen V. Oxytocin receptor gene (OXTR) polymorphisms and social, emotional and behavioral functioning in children and adolescents: a systematic narrative review. Neurosci Biobehavioral Reviews. 2022;135:104573. 10.1016/j.neubiorev.2022.104573 35149102

[9] de Oliveira Pereira Ribeiro L, Vargas-Pinilla P, Kappel DB, Longo D, Ranzan J, Becker MM, et al. Evidence for association between OXTR Gene and ASD clinical phenotypes. J Mol Neurosci. 2018;65(2):213–21. 29858823 10.1007/s12031-018-1088-0

[10] Waller R, Corral-Frías NS, Vannucci B, Bogdan R, Knodt AR, Hariri AR, et al. An oxytocin receptor polymorphism predicts amygdala reactivity and antisocial behavior in men. Soc Cogn Affect Neurosci. 2016;11(8):1218–26. 27036876 10.1093/scan/nsw042 PMC4967804

[11] Wu N, Li Z, Su Y. The association between oxytocin receptor gene polymorphism (OXTR) and trait empathy. J Affect Disord. 2012;138(3):468–72. 22357335 10.1016/j.jad.2012.01.009

[12] Kalyoncu T, Özbaran B, Köse S, Onay H. Variation in the oxytocin receptor gene is associated with social cognition and ADHD. J Atten Disord. 2019;23(7):702–11. 28478728 10.1177/1087054717706757

[13] Francis SM, Kim SJ, Kistner-Griffin E, Guter S, Cook EH, Jacob S. ASD and Genetic associations with receptors for oxytocin and Vasopressin-AVPR1A, AVPR1B, and OXTR. Front Neurosci. 2016;10:516. 27920663 10.3389/fnins.2016.00516 PMC5118619

[14] LoParo D, Waldman ID. The oxytocin receptor gene (OXTR) is associated with autism spectrum disorder: a meta-analysis. Mol Psychiatry. 2015;20(5):640–6. 25092245 10.1038/mp.2014.77

[15] Meyer M, Jurek B, Alfonso-Prieto M, Ribeiro R, Milenkovic VM, Winter J, et al. Structure-function relationships of the disease-linked A218T oxytocin receptor variant. Mol Psychiatry. 2022;27(2):907–17. 34980886 10.1038/s41380-021-01241-8 PMC9054668

[16] Ma SL, Tang NLS, Tam CWC, Lui VWC, Lam LCW, Chiu HFK, et al. A PIN1 polymorphism that prevents its suppression by AP4 associates with delayed onset of Alzheimer’s disease. Neurobiol Aging. 2012;33(4):804–13. 20580132 10.1016/j.neurobiolaging.2010.05.018 PMC2988914

[17] Dadds MR, Moul C, Cauchi A, Dobson-Stone C, Hawes DJ, Brennan J, et al. Polymorphisms in the oxytocin receptor gene are associated with the development of psychopathy. Dev Psychopathol. 2014;26(1):21–31. 24059750 10.1017/S0954579413000485

[18] Chen Y, Lu R, Zheng H, Xiao R, Feng J, Wang H, et al. The NFKB1 polymorphism (rs4648068) is associated with the cell proliferation and motility in gastric cancer. BMC Gastroenterol. 2015;15:21. 25888547 10.1186/s12876-015-0243-0 PMC4331381

[19] Albert FW, Kruglyak L. The role of regulatory variation in complex traits and disease. Nat Rev Genet. 2015;16(4):197–212. 25707927 10.1038/nrg3891

[20] Dadds MR, Moul C, Cauchi A, Dobson-Stone C, Hawes DJ, Brennan J, et al. Methylation of the oxytocin receptor gene and oxytocin blood levels in the development of psychopathy. Dev Psychopathol. 2014;26(1):33–40. 24059811 10.1017/S0954579413000497

