# Supplementary material for: Functional significance of some common oxytocin receptor SNPs involved in complex human traits
Source: BMC Mol Cell Biol. 2025 Jan 6;26:3. doi: 10.1186/s12860-024-00529-1 (PMC11705901; doi:10.1186/s12860-024-00529-1)

Supplementary Figures

Fig S1. Luciferase assay showed no significant difference between different alleles of (a) rs4686302 (p=0.065); (b) rs237887 (p=0.068) and (c) rs2254298 (p=0.33) in H4.

(a)


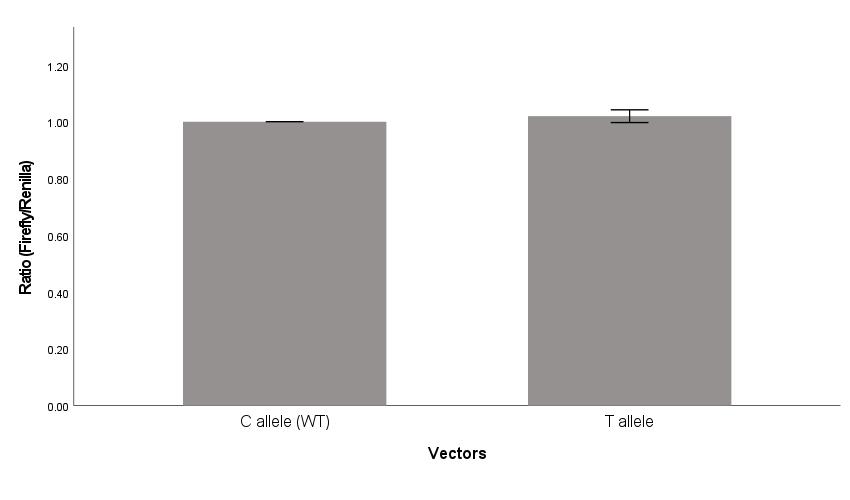


(b)


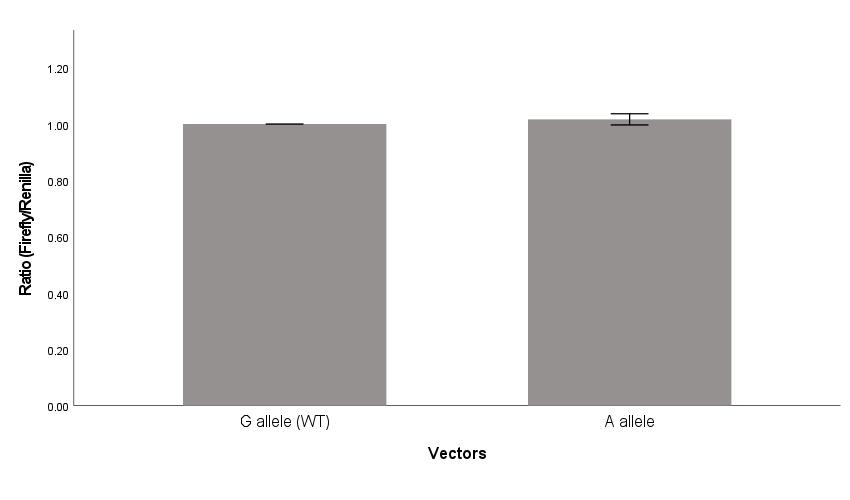


(c)


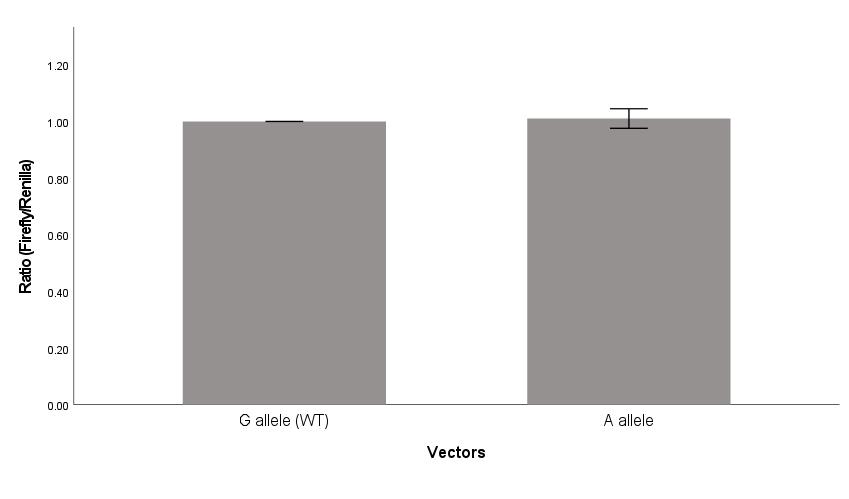

Supplement: Supplementary file 1 — Supplementary Material 1 [file 12860_2024_529_MOESM1_ESM.docx]
