# Supplementary material for: Functional significance of some common oxytocin receptor SNPs involved in complex human traits
Source: BMC Mol Cell Biol. 2025 Jan 6;26:3. doi: 10.1186/s12860-024-00529-1 (PMC11705901; doi:10.1186/s12860-024-00529-1)
Supplement: Supplementary file 3 — Supplementary Material 3 [file 12860_2024_529_MOESM3_ESM.docx]

Supplementary file showing the alignment of cloning vectors.

Bold base: rs237887

Highlighted sequence: Forward and reverse cloning primers

14041 g[cggaaggat tggtggctac t]taggaattc attaactatt ttcttctttg ttgttgatgt

14101 ttctgtagtt ttttaaaaaa taggataaaa aagttgaaag atccaaagag taaagagtga

14161 gaccaatagc ttaaatgaac agataagatt gtgccaaata actgtatcca actaggcaaa

14221 acagtgtctt tctgtgaact tcatccctga tgcttcct**c**c tacctcattg caaagcttta

14281 tacacctatg tccttccctt tgtggctggt aggttgtaaa tattgacttt tgcacgttga

14341 tttttgcctc tttcattatt tctaagacat tctggtttta gtcttgtggg cgaaccttcc

14401 ctttgtggct ggtaggttgt aaatattgac ttttgcacgt tgatttttgc ctctttcatt

14461 atttctgaga cattctgttt ttagtcttgt gggtgaattt caattttgtt ggtgaaatat

14521 tccatcagag tttctaaact taaattcatc ttcttatcca tccaaatcaa agcaaatttc

14581 ttaacaaaat attttttgtt aaaaccagtg aatttagtaa tattgatcat ttttctttca

14641 tttttcctcc tttctattct ttattatttt aatactaaga gttaaatttc cattctttgc

14701 tttctaaaaa ctctcttttg caagaatttt gttttctgtc tatcgtagct tgctcttgtt

14761 ccaacaggtg caccaattaa ttagactagt ggttcttaac cttgagggtg cattagaatt

14821 acaacattcc tgagccctac ctccagaggg taattctggg gtg[gggcctgagaatgtgca

14881 ttt]

Bold base: rs4686302

Highlighted sequence: Forward and reverse cloning primers

1261 tcacggccct cccggtttgt ttcag[ggtgg acccagcaga tccg]tccgtg gagtctccag

1321 gagtggagcc ccgggcgccc ctacaccctc cgacacgccg gatccggccc agccgcgcca

1381 agccgtaaag ggctcgaagg ccggggcgca ccgctgccgc cagggtcatg gagggcgcgc

1441 tcgcagccaa ctggagcgcc gaggcagcca acgccagcgc cgcgccgccg ggggccgagg

1501 gcaaccgcac cgccggaccc ccgcggcgca acgaggccct ggcgcgcgtg gaggtggcgg

1561 tgctgtgtct catcctgctc ctggcgctga gcgggaacgc gtgtgtgctg ctggcgctgc

1621 gcaccacacg ccagaagcac tcgcgcctct tcttcttcat gaagcaccta agcatcgccg

1681 acctggtggt ggcagtgttt caggtgctgc cgcagttgct gtgggacatc accttccgct

1741 tctacgggcc cgacctgctg tgccgcctgg tcaagtactt gcaggtggtg ggcatgttcg

1801 cctccaccta cctgctgctg ctcatgtccc tggaccgctg cctggccatc tgccagccgc

1861 tgcgctcgct gcgccgccgc accgaccgcc tggcagtgct cgccacgtgg ctcggctgcc

1921 tggtggccag cgcgccgcag gtgcacatct tctctctgcg cgaggtggct gacggcgtct

1981 tcgactgctg ggccgtcttc atccagccct ggggacccaa ggcctacatc acatggatca

2041 cgctagctgt ctacatcgtg ccggtcatcg tgctcgct**g**c ctgctacggc cttatcagct

2101 tcaagatctg gcagaacttg cggctcaaga ccgctgcagc ggcggcggcc gaggcgccag

2161 agggcgcggc ggctggcgat ggggggcgcg tggccctggc gcgtgtcagc agcgtcaagc

2221 tcatctccaa ggccaagatc cgcacggtca agatgacttt catcatcgtg ctggccttca

2281 tcgtgtgctg gacgcctttc ttcttcgtgc agatgtggag cgtctggg[atgccaacgcgc

2341 ccaagg]aagg

Bold base: rs2254298

Highlighted sequence: Forward and reverse cloning primers

8461 caactttggc actattgaca ttttaggc[tg aacagtcttt ggcgtgtg]tg tatgtgtgtc

8521 agggaggagc tgttctgtgc atggtaggat atttaacagc attcccgtcc tccacgtgct

8581 acgtgccatt agcaacctcc ctgccagctg tggccaccaa aactgtctcc agacattgcc

8641 aaaagttcac taaggggcaa agtcatccct ggttaagaac tgctggctta gaccccttgt

8701 tctgcttttt aaactaatac ctggcagtga gtactctctt gttgccctgg tctctggcca

8761 gcctgccttg tgtaccccca gccaggcccg atgatttgcc gctttccaca agttcctatg

8821 gccttgctgt ctttgcacct ttgctaatac tgttcctgcc atgggaacgc ccaccccagt

8881 ttcttccacg gattcccatc ctttgaagcc caagtccatt gtgtgggctg ctttgtggca

8941 caactccagg ggatgccttt tacctcatgg tctatgaaca gggtgttcct aggagctttg

9001 cagtgcacag cctgaacagc tgaacatggc agcctcatcc agtgcccctt tcaggaaacc

9061 atccctgttt tc**c**cagtttg cggggcttct tcctctgaat gctaagtcat tcactccaaa

9121 aaacagttac tgatgcctct gcaggctcct gtccacacaa cctctgcttt catctgtcaa

9181 cccccaagtg aagagagttg gcaaggggcc ctcccaagcc ctcgccctcc tgcggccagc

9241 tgctgacatg cataaatgtg ggaccgtgtg gtcaaggttg atcacagagt tctgggtaca

9301 cccacagacc tctgggaggg acacccacag tttctgctgg gttagtgtca gatggagcgg

9361 cagtgctgac ttcgggtgtg gggctgagcc tgcccacaac tcaggtttct ggggttcaga

9421 atctcaggtt tctgaagttc agaataggac ttaggttgta tacagggtgg aatatcctgc

9481 tggtgcaagt cagtgtagct gatggagtgg gagtca[tttt gggtacaggc ctagca]tttt

Bold base: rs1042778

Highlighted sequence: Forward and reverse cloning primers

16621 cagcca[tcca cggcgtgacc cacca]gccag ggccagggct gcagcctgag gctcaggctg

16681 tgctggcata agtgctctgc tcctaggtga tggcgtatgt ttgtgtataa ggtacctatc

16741 agtttgtatc cctcccctcc ttggggtggc ttcagtgggg tggagagtgg cctccatgat

16801 ggaagatgat aggggactca gccatcagac aacaccctgg cctcctacac gtacttctac

16861 caccctgaac ccactgctgc cctgggcagt gagtggcttg ttttttctcc tggacttgta

16921 atttcactcc agtatatttt tacttcttca ttctgggata ttgtgaaaag cggtaaatat

16981 aggattggtg accaattggg tcaggaagtc cagtgttctg gacttggggt aagcagtggg

17041 gttgggacct cagatgggaa gggtggtgct aagatcctcc tgacctcaaa gtgtatttgc

17101 ctttaagcga acaaatgctg gggtccttgg ggaccagctt gtcagagggt agccctaaga

17161 gaaggggatt accttgtaag accatctggc gcagtggacc tattagaact tgggttaaaa

17221 atgtttaaga agctaatgtt taagaagcat ttgggaaaga aaaagaaata aatgtatcca

17281 gataggaaaa gaagaagtaa aactatttgc agatgacaca gttttgtata tagaaaatcc

17341 taaggaactc acacacacac acacacacac acacgcacac agctattaga actaataagc

17401 aagttccgca aggtttcaag atacaagatc aatatacaaa aatgaattgt atttctttat

17461 actagcaaca aacaatatga aaacgaagtt aaataattcc atttataata ccatcagaaa

17521 gaataaaata ggaatcaact taacaaaaca agtgcaagac tgaaaactac aaaattggaa

17581 agaaattaaa gaaggcttaa ataaatggaa agacatcctg tgttcatgga tcagacttag

17641 tattgttaag atggcaatac tatcctaact gacatgcaga ttcagtgcaa tccttatgaa

17701 aatcatagct ggcttcttta cagaaattga taagctagtc ccaaaattca taaagaaatg

17761 caagggaccc agaatatcca aataagcctt gaaaaagaac aaagttggtg gattcacact

17821 tcctgatttc ataatttacg ataaaggtaa tcagctcagt gtgttactgg tttaaggata

17881 gacatacgga gcagaataaa gagtacagat atgaacactt atacttacgg tcaattgatt

17941 tttgacaagg ttcccaagac aattcaatag agaaaggaga gtcttttcaa caaatggcac

18001 cgagacaatg atatgcaagt gcaaaagaat gaggttggac ctttactcac actatgtgca

18061 aaaatcaact caaaacgcat ccaagatcta aatataagag ctgaaactat aaaatcttag

18121 aaagaaacat aggcatagat ctttgtaacc ttgaattagg cagtggtttc ttagatatga

18181 taccaaagac acaagcaacc aatggaaaaa taggtaaatt ggacttaatc aagatttgaa

18241 gcttttgtga ttgaaaagac cctatcaaga aggtgaaaag ataacctgca gaatgggaga

18301 aaatatttgc gagtcatata tatgataagg ggcttgtatc tggaatatat aaataactct

18361 tataacacaa caataaggag aaaaataaat caatttaaaa aatgggctaa cggtttgaat

18421 agacatttct ccaaagaaga tatgcaaatg gctactaagc acatgaaaaa atactcaaca

18481 ttattattca ttagggaaat gcaagtcaaa atcacaatga gattccagtt tacaatcact

18541 aggatggcta caataaaaag atggacaaga acgagtgtcg gtgaggatgt agagaaactg

18601 gtagaaattt aaattgttgg tgggaatgta aatggtgcac ctgctttgaa aaacagtttg

18661 gcagtacctc aaaaagttaa acgtagagtg accatatgac ccaggaatgc cactcctagg

18721 tatttaccca agagaaatga aaacgtacat acacacaaaa acttgtacac caatgttcat

18781 agcaacatta tttgtaatag ccaaaaagtg gaaacaaccc aaatgtctac caactgatga

18841 atgggaaata aaatgtggtc tgtccacgca atggaacatt attagactct aaaaagaaat

18901 gaagtactca cacatgccac aacatggatg agccttgaaa acttgctaag tgaaagaagc

18961 caggtgcaaa agcccacata ttgtctgact gcattgaaat gcaatgtcta aaatggacga

19021 atctatatag agtgaatata gattagcgtt tgccagggcc tggaggctgt gagagatgag

19081 gcatgactac taagggtttg gggtttcttt ttcgggtgat gaaaatgttc tgaaattagt

19141 ggtgattgtg cacgattttg agaatgtact aaaaaccaat [gaactttaaaaaataaaaat

19201 aaac]aaa
